# Case Report: Recurrent pediatric cavernous malformation of the trigeminal nerve

**DOI:** 10.3389/fsurg.2023.1278177

**Published:** 2023-12-22

**Authors:** Mikhail Harty, Muhammad Waqas Saeed Baqai, Jahangir Sajjad, Greg Fellows, Philip J. Clamp, Kumar Abhinav

**Affiliations:** ^1^ Department of Neurosurgery, Southmead Hospital, North Bristol NHS Trust, Bristol, United Kingdom; ^2^ Department of Paediatric Neurosurgery, Bristol Royal Hospital for Children, University Hospitals NHS Trust, Bristol, United Kingdom

**Keywords:** cranial nerve cavernoma, cavernous malformation, trigeminal nerve cavernous malformation, pediatric, cavernoma

## Abstract

**Background:**

Most cavernous malformations (CM) usually involve the parenchyma and rarely occur in cranial nerves. Recurrence of CM associated with cranial nerves after surgical resection has not been previously reported.

**Case description:**

This paper describes the case of an 11-year-old girl who presented with left otalgia and headache because of a left trigeminal cavernous malformation. She underwent radical resection via a left retrosigmoid approach while sparing the trigeminal nerve. Surveillance imaging at 18 months demonstrated recurrence along the length of the trigeminal nerve into Meckel's cave with significant extension into the middle cerebellar peduncle. Subsequent re-operation via an extended middle fossa approach with anterior petrosectomy enabled complete resection with division of the trigeminal nerve. Postoperatively, she had a transient left facial paresis, and right hemiparesis that resolved within 48 h.

**Conclusion:**

This case highlights the importance of close postoperative surveillance in CM associated with cranial nerves as recurrence after nerve-sparing resection is possible. Surgical treatment due to the morphology of significant recurrence required the use of a complex skull base approach through a new corridor to achieve optimal clinical outcome.

## Introduction

Cavernous malformations (CM) are relatively common vascular malformation that occurs in 0.9% of the general population (1). Typically, they are found within the brain parenchyma while involvement of cranial nerves is rare (2–4). Gross et al. found that in persons less than 21 years, CM present with an average age of 10 years (5). To our knowledge we present the first case of a pediatric case with recurrent CM along the anatomical distribution of the trigeminal nerve and the surgical strategy to address the recurrence.

## Case report

**Figure 1:**
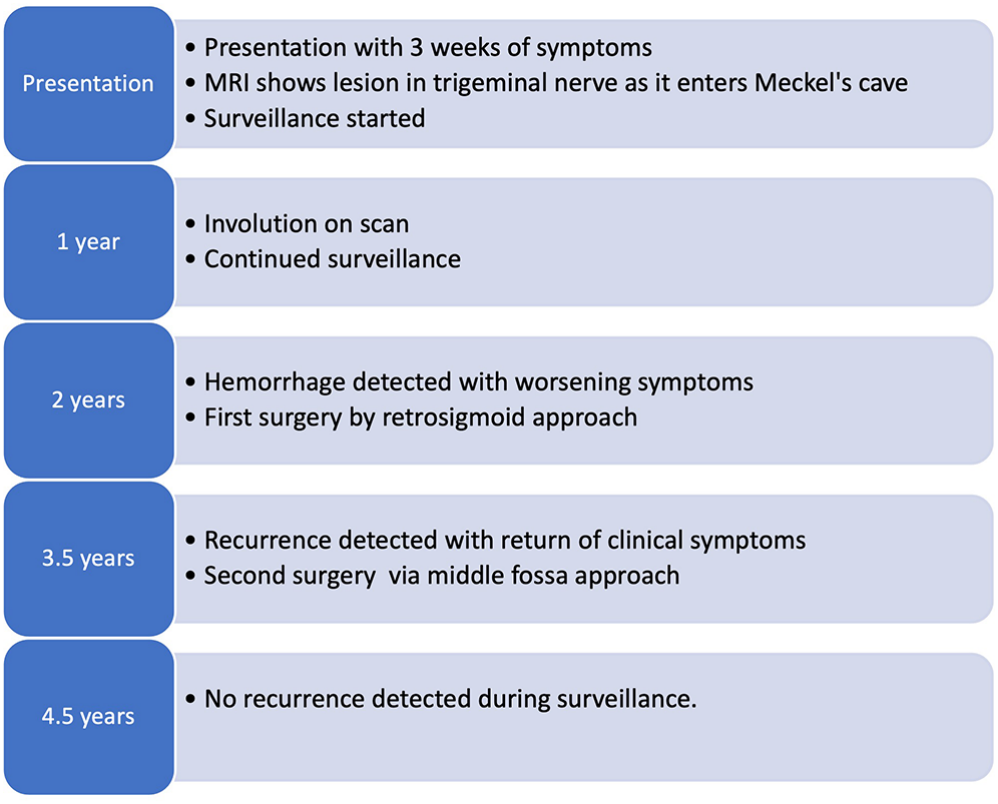
Schematic representation of the timeline from presentation and primary surgery through the recurrence of the lesion and the second operation.

**Figure 2:**
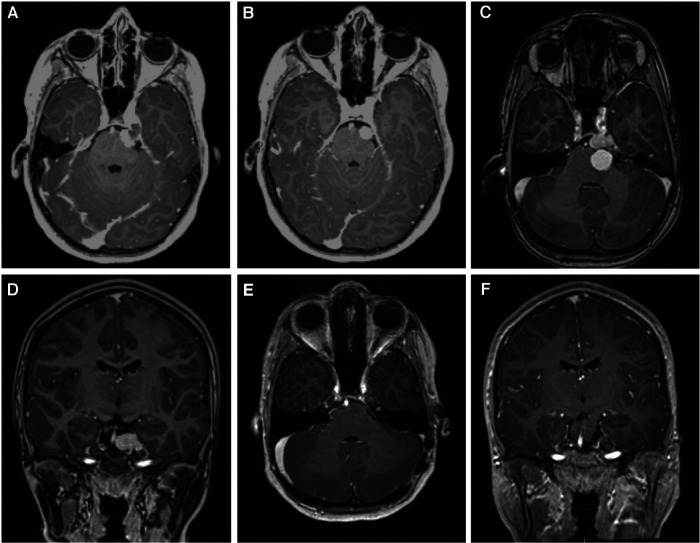
(**A**) Axial contrast enhanced T1-weighted image showing a lesion in the left trigeminal nerve extending from pontine portion into Meckel's cave. (**B**) shows interval image at 1 year after presentation which showed involution of the lesion. (**C, D**), show new hemorrhage with cystic portion invaginating the brainstem which prompted surgical intervention. (**E, F**) show 6-month postoperative contrast enhanced T1-weighted axial and coronal images, respectively, after the first operation via retrosigmoid approach demonstrating gross total resection.

An 11-year-old girl presented with a 3-week history of left otalgia, headache, and an episode of syncope. Examination revealed normal neurologic findings and no focal deficits (Figure 1). Magnetic resonance imaging (MRI) of the brain showed a hemorrhagic cystic lesion in the left cerebellopontine angle (CPA) with a differential diagnosis of schwannoma vs. trigeminal CM. She initially underwent a period of surveillance during which time the lesion involuted at 1 year (Figure 2). At 2 years, repeat images showed new hemorrhage and the decision was made for surgical intervention. She underwent a left retrosigmoid craniotomy and was found to have a CM embedded within the cisternal segment of the trigeminal nerve that extended into Meckel's cave. A satisfactory resection was thought to have been achieved at surgery with preservation of the nerve fascicles and on postoperative imaging. She had an unremarkable postoperative course.

**Figure 3:**
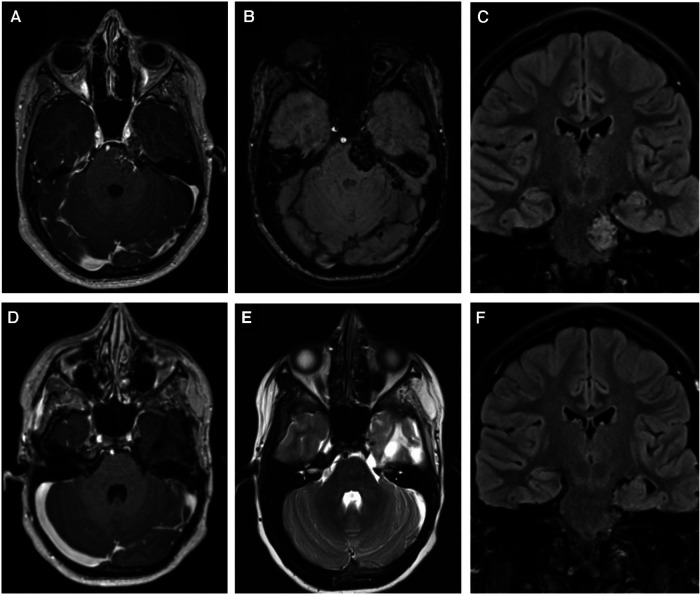
Shows images before and after the second operation via a subtemporal Kawase-Dolenc approach. (**A**) contrast enhanced T1-weighted imaging and (**B**) Susceptibility weighted image demonstrate the lesion filling Meckel's cave extending along the cisternal segment of the trigeminal nerve and invaginating the brain. (**C**) demonstrates the coronal FLAIR that shows the CM involving the brainstem and extending down to the internal acoustic meatus. (**D,E**) are contrast-enhanced T1, and T2-weighted images, respectively, and (**F**) is a coronal FLAIR that demonstrate complete resection of the recurrent lesion.

Imaging done up to 12 months after surgery demonstrated no recurrence of the lesion. By 18 months she began to experience significant left-sided facial hypesthesia and sensory symptoms worse in the distribution of the 3rd division of the trigeminal nerve. MRI revealed considerable recurrence of the lesion along the anatomical extent of the trigeminal nerve which distorted the ventrolateral brainstem, middle cerebellar peduncle (MCP) and extended into the Meckel's cave and caudally below the trigeminal nerve toward the internal acoustic canal (Figure 3).

**Figure 4:**
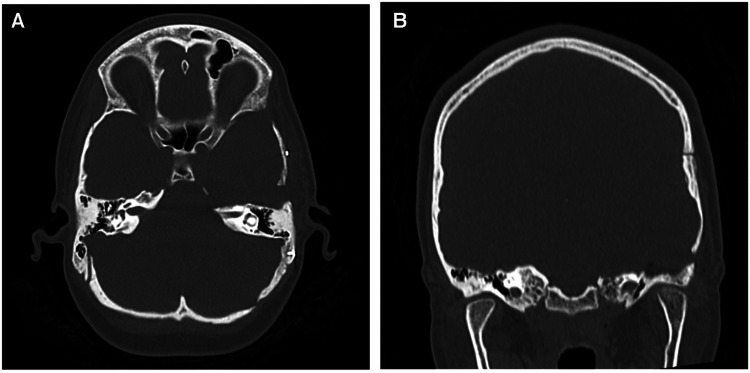
(**A**) axial and (**B**) coronal CT images demonstrating the extent of the anterior petrosectomy to give access to the lesion during the Kawase-Dolenc approach at the second operation.

The second operation utilized an extended middle fossa (Kawase-Dolenc) approach. Intraoperative neuromonitoring was employed to aid resection. Key operative steps included a frontotemporal craniotomy with an anterior interdural approach to the mandibular nerve then posterior dural elevation to undertake anterior petrosectomy (Figure 4). The large solid exophytic CM appeared intimately related to the anatomical trajectory of the trigeminal nerve with a significant component embedded within the brainstem. The brainstem component was gently removed in piecemeal fashion while respecting the hemosiderin-stained parenchyma. The trigeminal nerve was sacrificed to achieve gross total resection with the tentorial division and mobilization to further permit the removal of the component within Meckel's cave. Postoperatively, she had a transient left facial paresis and right hemiparesis resolving within 48 h. A small pseudomeningocele resolved within four months. She had facial hypesthesia that persisted after the operative intervention.

Postoperative images showed gross total resection with no evidence of radiological recurrence at 12 months (Figure 2). Genetic testing for familial CM was negative for mutations.

## Discussion

Trigeminal CM are extremely rare with only 17 cases reported in literature (2, 4, 6–19). Majority of these cases presented with trigeminal neuralgia and were treated with surgical resection. Only 2 were treated with stereotactic radiosurgery and one with percutaneous balloon compression (10, 13, 19). Adachi et al. classified these lesions into 4 types according to the origin of the CM: within the Gasserian ganglion, within the cisternal segment, within the intra-axial trigeminal nerve root in the pons, and within the spinal tract of the trigeminal nerve.^6^The retrosigmoid approach was chosen for the first operation because it gave access to the cerebellopontine angle and is a workhorse approach for this region (4, 20). This approach facilitated removal of the hemorrhagic cystic component invaginating the brainstem while allowing extirpation of the CM. Gross total resection was thought to have been achieved intraoperatively with preservation of the trigeminal nerve fascicles and was confirmed with postoperative surveillance MRI.

The decision-making process concerning the management of the recurrence took into consideration that the lesion was high risk for clinically significant re-hemorrhage due to at least two previous documented radiological evidence of hemorrhage before the first surgery. Moreover, there was now a significant large volume symptomatic recurrence over a short period of time after prior surgical resection. Stereotactic radiosurgery was deemed to be feasible and was offered. However, due to the factors as outlined above in addition to the favorable exophytic nature of the lesion, after discussion with the family, surgery via a new operative corridor was undertaken.

At the second operation, a different anterolateral operative corridor was chosen to avoid the scarred retrosigmoid corridor and, more importantly, to resect the lesion along the axis of its recurrence while avoiding neural transgression of the surrounding middle cerebellar peduncle. In addition, the extended middle fossa approach with anterior petrosectomy enabled access to the ventrolateral pontine surface. This allowed the caudal extent of the recurrence below the trigeminal nerve to be safely resected while ensuring removal of the component within Meckel's cave after tentorial division to achieve gross total resection.

Recurrence after gross total resection of CM in the pediatric population has been rare with a reported rate of 0.7% at 1 year but none of these described recurrence of CM associated with a cranial nerve (21). Samadian et al., in a case of CM associated with the trochlear nerve recommended sectioning and reanastomosis of the nerve to achieve resection (8, 22). Liu et al. in their case report described a CM of the trigeminal nerve that was intimately related to the sensory root which was sacrificed while preserving the motor root (11). Mascarenhas et al., described resection of a CM adherent to the second and third division of the trigeminal nerve that required taking some of the rootlets of the nerve (12). It is likely in our case that the CM was located within the trigeminal nerve substance and a small remnant along the fascicles facilitated the recurrence after the first surgery while attempting preservation of the nerve. At the second operation, due to significant recurrence and because of the intimate relationship between the CM and the trigeminal nerve, the preservation of the nerve could not have been undertaken in any meaningful manner and therefore it was sacrificed during the CM resection. Attention was also paid to remove the Meckel's cave component to reduce the risk of recurrence.

## Conclusion

Close clinical and radiological surveillance after surgical resection of trigeminal or a cranial nerve CM is needed because the clinical course is ill-defined in the pediatric population with a potentially higher risk of recurrence (9, 21). This case additionally highlights the importance of the use of complex skull base approaches when required to achieve optimal clinical and surgical outcome in patients with surgically challenging recurrent CM particularly for those that directly or indirectly involve the brainstem.

## Data Availability

The raw data supporting the conclusions of this article will be made available by the authors, without undue reservation.
